# Expression of PTEN‐long mediated by CRISPR/Cas9 can repress U87 cell proliferation

**DOI:** 10.1111/jcmm.13236

**Published:** 2017-06-19

**Authors:** Na Fang, Tingxuan Gu, Yahui Wang, Shuzhen Wang, Fengling Wang, Yang An, Wenqiang Wei, Weijuan Zhang, Xiangqian Guo, Adil J Nazarali, Shaoping Ji

**Affiliations:** ^1^ Department of Biochemistry and Molecular Biology Medical School Henan University Kaifeng Henan Province China; ^2^ Jiangsu Superbio Co.,Ltd Nanjing China; ^3^ College of Pharmacy and Nutrition and Neuroscience Research Cluster University of Saskatchewan Saskatchewan Canada; ^4^ Department of Oncology The First Affiliated Hospital of Henan University Kaifeng China

**Keywords:** PTEN‐long, CRISPR/Cas9, gene edition, gene therapy

## Abstract

PTEN is a tumour suppressor that is frequently mutated in a variety of cancers. Hence, PTEN has significant potential as a therapeutic molecule. PTEN‐long is an alternative translation variant, with an additional 173 amino acids added to the N‐terminal of the canonical PTEN when CUG of the mRNA is utilized as the start codon. PTEN‐long is secreted into serum and can re‐enter cells throughout the body. One of the major barriers for gene therapy is to efficiently and specifically deliver DNA or RNA material to target cells. As an alternative approach, if a therapeutic protein can be directly delivered to target cell of interest, it should theoretically function well within the cells, particularly for genes that are deficiently expressed *in vivo*. Most therapeutic proteins are incapable of efficiently permeating the cell membrane. In this study, we have employed CRISPR/Cas9 gene editing tool combined with single‐stranded template to edit CTG of PTEN‐long to ATG in the genome. Two guide RNAs close to CTG site were found to have similar efficiency in driving PTEN‐long expression. Furthermore, we detected PTEN‐long expression in transfected whole‐cell lysate and in concentrated culture media in Western blot. Interestingly, the culture media of PTEN‐long expression can reduce Akt phosphorylation level and repress U87 cell proliferation compared to wild‐type U87 or control media. Taken together, PTEN‐long driven by CRISPR/Cas9 imports and exports cells and represses nearby cell proliferation, indicating the PTEN‐long generated by CRISPR/Cas9 has potential to be an alternative strategy for PTEN gene therapy.

## Introduction

CRISPR/Cas9 is a novel gene editing tool derived from prokaryotic adaptive immuno‐system against phage/virus and foreign DNA invasion. It is widely used in mammalian genomic editing, including repressing gene expression 1, gene knock‐in 2, repairing disease‐related genes 3 and development of animal models of cancer 4. Increasing evidence suggests that CRISPR/Cas9 may play an important role as a tool in gene therapy for human genetic diseases 5, 6, 7, particularly with recent investigation of pathogenic gene editing in animals 8, 9, 10. Technically, a guide RNA is designed to recognize the target DNA sequence and the Cas9 enzyme induces a double‐stranded break on the DNA of interest. At the same time, a template DNA with a corrected/mutated sequence is required to edit/repair the target DNA through endogenous homology‐directed repair (HDR) activity 11, 12. However, in the absence of the DNA template, the broken DNA could reconnect through a non‐homologous end joining (NHEJ) 11, which may induce a mutation *via* a deletion or insertion of base pair (s)^13^. Subsequently, a Cas9 nickase, inducing a nick on double‐stranded DNA, was engineered to increase the number of specifically recognized bases and reduce off‐target cleavage 14.

Recently, a class 2, type VI CRISPR–Cas effecter C2c2 was identified and subsequent investigations indicated it can cleave single‐stranded RNA 15. Thus, modification /alteration of CRISPR–Cas extended its utilities in editing of nucleic acid from DNA to RNA. For genomic editing, this method is primarily used to repair a DNA sequence of short span 11, where HDR may easily be carried out. In this study, we employed classic CRISPR/Cas9 to edit only one base pair on genome at HEK293 cell line, to induce expression of a PTEN variant (PTEN‐long).

PTEN (Phosphatase and tensin homolog) is a phosphatase that dephosphorylates phosphatidylinositol trisphosphate (PIP_3_) to PIP_2_ and down‐regulates PI3K‐Akt signalling, which plays a critical role in cell proliferation and tumorigenesis 16. PTEN is one of the most frequently mutated gene in a variety of cancers 17. Recent investigation revealed that PTEN has an extended translation variant, PTEN‐long, that is alternatively translated from the upstream of canonical PTEN mRNA with CUG as start codon 18. PTEN‐long has additional 173 amino acids added to N‐terminal of the canonical PTEN. It has also been verified that PTEN‐long is able to negatively regulate PI3K‐Akt pathway activity much like the canonical PTEN activity 18. PTEN‐long has a low expression level but can be secreted in paracrine manner into plasma and impact neighbouring cells or impact distant cells *via* the circulatory system 19. The ability of PTEN‐long to be exported and imported into cells confers its potential use in gene therapy as a substitute for canonical PTEN. Considering the difficulty of delivering a therapeutic vector to target cells in gene therapy, PTEN‐long has the advantage that it can be efficiently delivered to anywhere in human body *via* the circulation. An important advantage would be that PTEN‐long possesses all of the same amino acid sequence as endogenous protein and can thus avoid risks of immunogenicity.

Researchers have attempted to repress cancer proliferation with PTEN gene delivery to cancer tissues *via* vectors. The suppressive effect on cell proliferation by PTEN was measured for several different cancers, but findings were not as expected 20, 21. Recently, repression of PTEN expression mediated *via* CRISPR/Cas9 was carried out in mouse liver which induced a significant decrease in PTEN expression 22. These results suggest that CRISPR/Cas9 is able to efficiently edit PTEN gene *in vivo* to alter expression of PTEN. In this study, we used CRISPR/Cas9 combined with editing DNA template to target the start codon CUG of PTEN‐long to increase PTEN‐long expression. After transfection, codon alteration of CTG/CUG to ATG/AUG was identified, which significantly increased PTEN‐long translation compared to the original CUG codon of PTEN mRNA. It has been reported that the CUG codon compared to AUG start codon is less efficient at initiation of a protein translation 23, 24. Our findings show that as a result of change of start codon from CUG to AUG, this significantly promotes PTEN‐long expression. Similar to endogenous PTEN‐long, CRISPR/Cas9‐created PTEN‐long only has one amino acid change, the first leucine to a methionine. PTEN‐long protein was detected in both the cell lysate and cultured media. Additionally, we also report that the culture medium from the edited cells is capable of inhibiting U87 (PTEN‐null) cell proliferation.

## Materials and methods

### RNA‐guided plasmid construction

Two gRNA sequences against PTEN locus were designed with the use of CRISPR Design tool (http://crispr.mit.edu) from MIT. Both oligo DNA fragments encoding for gRNA were synthesized by Sangon Biotech™ (Shanghai, China). The two complementary DNA fragments were annealed to form a double‐stranded DNA segment bearing sticky ends compatible with GeneArt CRISPR Nuclease Vector (Invitrogen™ Carlsbad, CA, USA). This vector produces a fusion protein containing a self‐cleaving site 2A, in which Cas9 protein and the orange fluorescence protein (OFP) are separately released after translation. The construct was developed by ligating the two DNA segments into the GeneArt CRISPR Nuclease Vector, respectively. The DNA ligation product was transformed into TOP10 chemical competent *E. coli* and the inserted gRNAs were verified by DNA sequencing with U6 promoter primers according to the manufacturer's instructions.

### Development of pcDNA PTEN‐long expression vector

Human HEK293T cells were cultured as described 25. Total RNA was extracted from these cells and mRNA reverse‐transcribed to cDNA with 18‐nt poly‐T primer. PTEN‐long cDNA was amplified by PCR with an upstream primer carrying a mutation (CTG to ATG). Upstream primer: 5′‐GGATCCATGGAGCGGGGGGGAGAAG‐3′ and downstream primer containing a stop codon: 5′‐ GAATTCTCAGACTTTTGTAATTTGTGTA‐3′ were used. Following PCR amplification, the DNA product was purified and ligated into pGEM T‐easy (Promega™ Madison, WI, USA) vector and the coding region verified by sequencing. The DNA product with the correct coding sequence was subcloned into pcDNA3.1myc‐his‐A for cell transfection.

### Design and synthesis of ssODN templates

The 120‐nt ssODN (single‐stranded oligonucleotides) repair template (HPLC purified; Sangon Biotech™) was designed with homologous genomic flanking sequence around the PTEN‐long start codon CTG with only a single nucleotide C mutated to A (shown in bold below). The ssODN serves as the donor template sequence that can repair the double‐strand break occurring at PTEN‐long start codon through HDR within the cells. The ssODN sequence: 5′‐AAGGTGGAAGCCGTGGGCTCGGGCGGGAGCCGGCTGAGGCGCGGCGGCGGCGGCGGCACCTCCCGCTCATGGAGCGGGGGGGAGAAGCGGCGGCGGCGGCGGCCGCGGCGGCTGCAGCTC‐3′

### Cell transfection and collection of the culture medium

HEK293T cells and U87 cells were cultured in DMEM medium with 10% FBS at 37°C and 5% CO_2_, 100 μ/ml penicillin and 0.1 mg/ml streptomycin. One day before transfection, 6 × 10^5^ cells were seeded into 10‐cm dishes in culture media without antibiotics. At~70% confluency, HEK293T cells were transfected with 3 μg of CRISPR nuclease vector construct and 1 μg of ssODN DNA or pcDNA PTEN‐long expression vector with Biotool™ transfection reagent according to manufacturer's protocol. To reduce occurrence of NHEJ and facilitate HDR, 1.0 μM of DNA ligase IV inhibitor, SCR7 (Selleck™, Houston, TX, USA), was simultaneously added with the transfection 12, 26. The medium was changed with regular culture medium containing 1.0 μM of SCR7 12 hrs following transfection. The cells were examined and images captured under epifluorescence microscope after 24 hrs transfection at 548‐nm wavelength. Images at 10× and 20× magnification were taken under epifluorescence microscope or visible light, respectively. The cells bear orange fluorescence protein were separated from other cells by fluorescence‐activated cell sorting. To obtain secreted PTEN‐long, culture media of the cells transfected with CRISPR/Cas9 were collected 36 and 72 hrs, respectively, after transfection. Isolated culture media were concentrated (×50) by ultracentrifugation to increase protein concentration for detection with Western blot analysis.

### Genomic DNA extraction

The CRISPR/Cas9‐transfected HEK293T cells were cultured for 48 hrs or longer. Cells (1 × 10^6^) were harvested by trypsin–EDTA and washed with PBS by centrifugation; 600 μl of TNES buffer (10 mM Tris‐HCl, pH7.5, 400 mM NaCl, 100 mM EDTA, 0.6%SDS) with 35 μl of freshly prepared Protease‐K (20 mg/ml) was added to the cell pellet, and this was allowed to incubate overnight at 50°C. This was followed by the addition of 166.7 μl of 6M NaCl and samples were mixed. The samples were subsequently centrifuged at 12,000 *g* for 10 min. at room temperature and supernatant carefully removed into new tubes. Ice‐cold ethanol (800 μl) was added to the supernatants with gentle mixing. The samples were centrifuged at 12,000 *g* for 10 min. at 4°C and supernatant was discarded. The remaining DNA pellets were washed twice in 700 μl of anhydrous ethanol by centrifugation and samples allowed to dry for 10 min. DNA samples were re‐suspended in 200 μl of water and used for PCR or kept at −20°C for later use.

### Genome PCR and Sequencing

The genomic DNA from both transfected cells and untransfected cells was amplified by PCR. Forward PCR primer: 5′‐CAGGCGAGGGAGATGAGAGAC‐3′ and reverse primer: 5′‐AATCCTCCGAACGGCTGCCTC‐3′ were used. The genomic DNA was amplified by PCR with an initial pre‐denaturation at 98°C for 2 min., followed by 30 cycles of: 95°C for 40 sec.; 55°C for 40 sec.; 72°C for 40 sec.; ending with 72°C for 5 min. The PCR products were analysed in 1% of agarose gel and purified with a gel extraction kit (Omega™ Norcross, GA, USA). The amplified DNA was cloned into pGEM T‐easy vector (Promega™), according to manufacturer's protocol. Ten clones were isolated and analysed by EcoR I digestion. All positive clones were identified by Sanger sequencing (Sangon Biotech™).

### Western blot analysis

Both transfected and untransfected HEK293T cells and U87 cells were harvested by trypsin and lysed in RIPA buffer (20 mM Tris‐Cl (pH 7.5), 150 mM NaCl, 1.0 mM EDTA, 1% NP‐40, 0.1% SDS and 0.1% sodium deoxycholate) on ice for 30 min. in the presence of protease inhibitors (Sigma‐Aldrich™ Santa Clara, CA, USA) or 1.0 mM of activated sodium orthovanadate. The lysates were centrifuged 12,000 g for 15 min. at 4°C followed by addition of 4× SDS‐loading buffer (200 mM Tris‐Cl (pH 6.8), 400 mM DTT, 8% SDS, 4.0 mM EDTA, 0.4% bromophenol blue, 40% glycerol) to the supernatant. The supernatant samples were boiled at 100°C for 5 min. and centrifuged 12,000 *g* at room temperature for 10 min. The protein samples were analysed *via* SDS‐PAGE electrophoresis, following transfer of protein onto a PVDF membrane (Millipore™, Billerica, MA, USA). The membranes were probed with anti‐PTEN antibody (9188, Cell Signaling Technology™ Danvers, MA, USA), anti‐Akt and phosphorylated AKt antibodies (Cell Signaling) and anti‐GAPDH (ProteinTech™, Wuhan China) antibody. A secondary goat anti‐rabbit IgG HRP‐conjugated (Boster™, Wuhan, China) antibody was employed to visualize bands with the ECL detection kit (Biotool™, Houston, TX, USA) and FluorChem E (Protein Simple™). Cells transfected with pcDNA PTEN‐long expression vector were collected for Western blot analysis after 24‐hrs transfection as described above.

### Cell proliferation assay

U87 cells were seeded on 96‐well plates at 1 × 10^3^cells per well for basic line number. Cells were divided into four groups with each group containing three equal wells. The first group of cells cultured in normal medium served as control; the second group of cells cultured in 50% of normal medium plus 50% of HEK293 cell culture medium served as the untransfected control. The third cell group was cultured in 50% of normal medium plus 50% of PTEN‐long‐expressing HEK293 cell culture medium and the fourth group with 25% of PTEN‐long‐expressing HEK293 cell culture medium. The media were refreshed every 24 hrs. Cell counts were recorded using a hemocytometer every 24 hrs for successive 5 days The mean cell counts for the three independent wells for each group were collected and cell proliferation curves plotted with GraphPad Prism 5.

## Results

**Figure 1:**
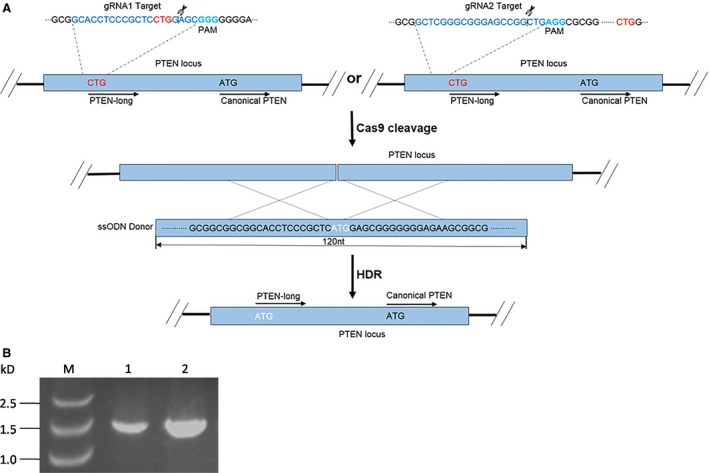
Development of CRISPR/Cas9 vector construct and the experimental process. (**A**) To screen and compare the efficiency of HDR occurring at different sites, two guide RNAs were designed using the nucleotide sequences as indicated. In order to facilitate homologous recombination repair after double DNA strand break, both cut sites were close to CTG which is a start codon for PTEN‐long translation. Two corresponding DNA segments derived from the guide RNAs were synthesized and ligated into GeneArt CRISPR Nuclease Vector. In addition, a 120‐nt single‐stranded DNA fragment was commercially synthesized and cotransfected with the vector into HEK293T cells. (**B**) To confirm whether the full‐length mRNA of PTEN is present in the HEK293T cells, PCR was carried out with primers covering PTEN‐long coding region. M: DNA ladder; Lanes 1 and 2 are PCR results from cDNA of HEK293 and HEK293T cells, respectively.

PTEN‐long is a PTEN translation variant, with an additional 173 amino acids at the N‐terminal of the canonical PTEN. The codon CUG within upstream of PTEN mRNA serves as start codon in an unclear mechanism to synthesize PTEN‐long protein. It is widely accepted that CUG as start codon is much weaker than AUG in the translation process 23, 24, 27. To promote PTEN‐long expression *in vivo*, we mutated codon CTG to ATG in the genome by employing CRISPR/Cas9 editing tool. In this study, two DNA sequences around the CTG start codon site were selected as the guide RNA sequences and cloned into CRISPR/Cas9 vector. Additionally, we synthesized a 120‐nt single‐stranded DNA fragment with C (TG) to A (TG) mutation (white letters in Fig. 1), which serves as the template in HDR process following transfection. As the Cas9 enzyme induces a break in the double‐stranded DNA mediated by guide RNA, the single‐stranded DNA is used as template to repair the broken DNA by HDR (Fig. 1).

**Figure 2:**
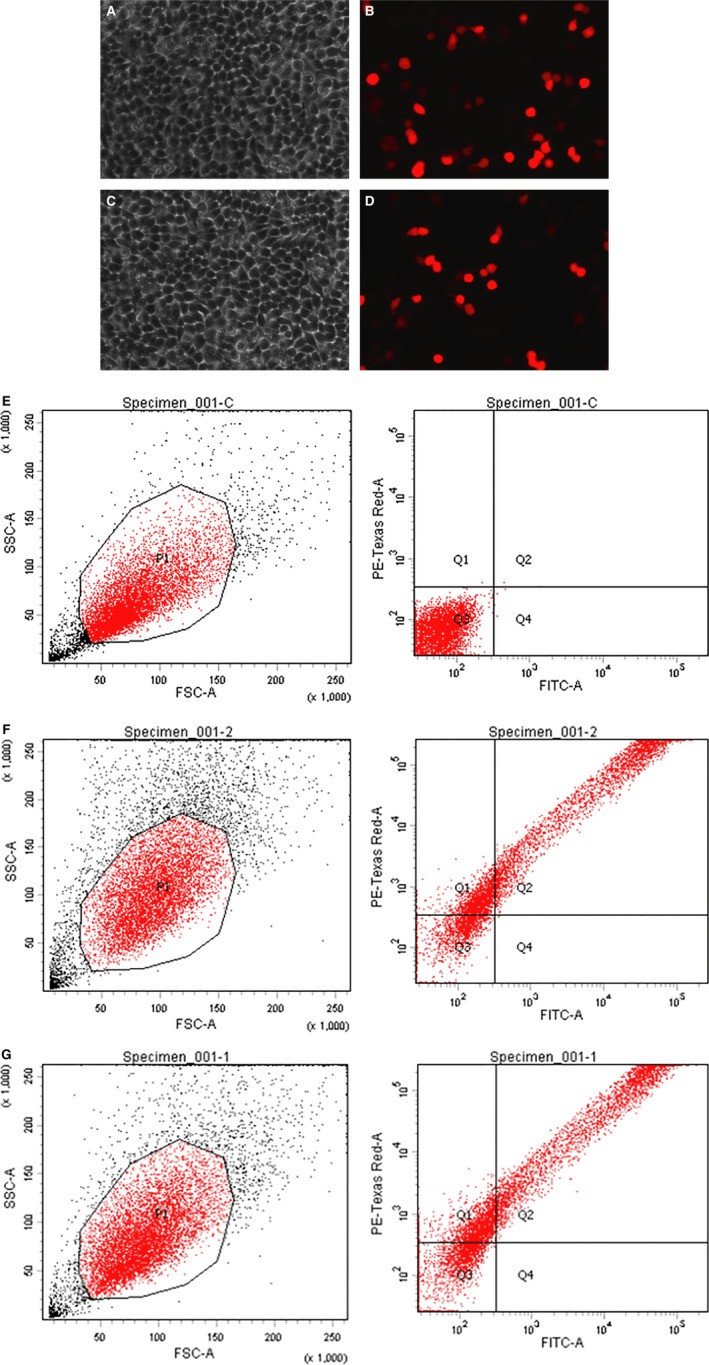
Transfection of HEK293T cells with CRISPR/Cas9. **A,C**. Cells were transfected with the vector containing guide RNA1 and guide RNA2 single‐stranded DNA, respectively. The images (20X) were taken 36 hrs after transfection. Images (20X) of cells under fluorescence microscope from guide RNA1 (**B**) and guide RNA2 (**D**). **A** and **B**, or **C** and **D** are the same view. The orange colour fluorescence protein was released from Cas9 *via* self‐cleaving site **A**. **E–G**, transfected cells were separated from other cells with fluorescence‐activated cell sorting, and transfection efficiency was calculated through flow cytometer. **E**, untransfected HEK293T cells; **F**, HEK293T cells were transfected with blank CRISPR nuclease vector; **G**, HEK293T cells were transfected with CRISPR nuclease vector plus ssODN DNA. P1:total cell number; Q1+Q2+Q4 = positive cell number; Q3 = negative cell number.

**Figure 3:**
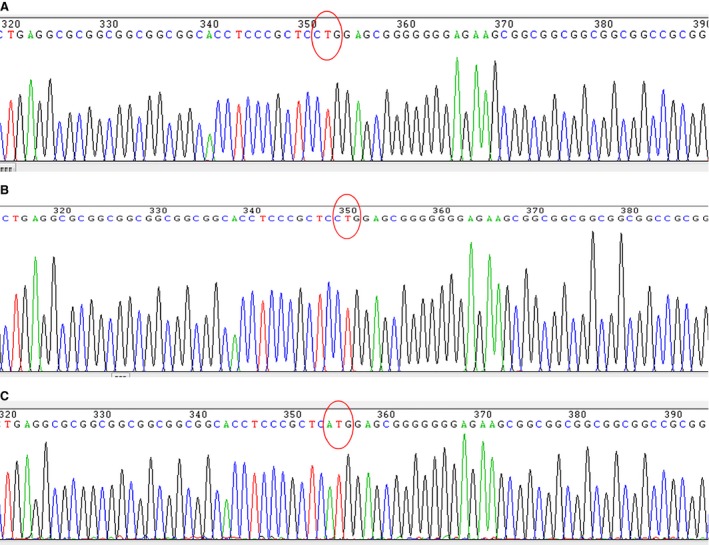
Genome sequencing of PTEN. Top panel: DNA sequencing from genomic DNA of the intact HEK293 cells. CTG in red circles start codon of PTEN‐long translation. Middle panel: Clones from PCR product from the transfected HEK293 cells indicate that HDR did not occur or DNA was from cells that remained untransfected with either the vector and/or single‐stranded DNA fragment. CTG shown red circle has remained. Bottom panel: CTG was successfully mutated to ATG indicated by the red circle, confirming that HDR had occurred within the cells after transfection.

**Figure 4:**
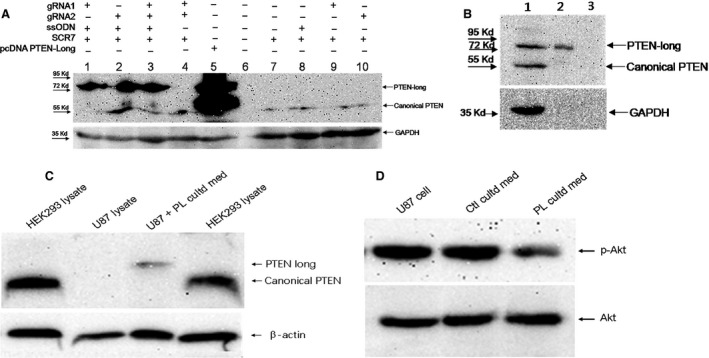
PTEN‐long exports and imports cells and inhibits Akt phosphorylation. **A**. The start codon CUG of PTEN‐long was mutated to AUG and PTEN‐long expression is significantly increased. gRNA1 and gRNA2 have similar efficiency in driving PTEN‐long expression (lanes 1 and 2). Combined gRNA1 and gRNA2 (lane 3) did not enhance PTEN‐long expression compared to lane 1 and 2. The ssODN is required for mutation of CTG to ATG through HDR and without which PTEN‐long is not expressed (lane 4). PTEN‐long cDNA cloned into pcDNA3.1 with CTG to ATG mutation was highly expressed in transfected HEK293T cells (lane 5). Lane 6 is protein ladder. Lanes 7–10 indicate that the combination of gRNA and ssODN is required to facilitate HDR occurrence and double‐strand DNA break. **B**. PTEN‐long can be secreted into cultured medium. Lane 1: whole‐cell lysate with PTEN‐long expression from transfected HEK293T cells. Lane 2: Cultured media concentrated (× 50) through ultrafiltration. Lane 3: Untransfected cell‐cultured medium. **C**. The concentrated PL cultured medium was added into U87 cell‐cultured medium, and then, the PTEN‐long was detected in the U87 lysate compared to intact U87 cell lysate or intact HEK293 lysate. **D**. concentrated PL cultured medium can reduce Akt phosphorylation level within U87 cells compared with control cultured medium or intact U87 cells.

HEK293T cells are derived from the human embryonic kidney (ATCC^®^ CRL‐1573™). These cells were co‐transfected with CRISPR/Cas9 vector combined with the single‐stranded DNA fragment. Following transfection, cells that are effectively transfected with CRISPR/Cas9 vector are visible under fluorescence microscope with the appropriate wavelength (Fig. 2). Orange fluorescence protein expressed by the vector can be separated from Cas9 protein by 2A self‐cleaving peptide and Cas9 protein retains its activity. The transfection efficiency is determined with flow cytometer. Both blank vector and guide RNA combined with ssODN reached approximately 70% transfection efficiency. The cells bearing OFP were separated from negative cells by fluorescence‐activated cell sorting. Prior to transfection, DNA sequence of the flanking regions with CTG target was characterized. A DNA fragment of 516 bp was amplified by PCR and verified by sequencing (Fig. 3). In addition, we examined and confirmed that the HEK293T cells express PTEN canonical protein and full‐length PTEN‐long mRNA (Figs 3 and 4).

Genomic DNA from the cotransfected HEK293T cells was extracted, cloned and analysed by DNA sequencing. Ten positive clones were identified by EcoR I digestion, and these were subjected to Sanger DNA sequencing. From these, three clones were identified to carry an alteration of CTG to ATG on the target site within PTEN gene (Fig. 3C), compared to intact cells or cells that were not effectively processed by HDR (Fig. 3A and B). DNA sequencing results indicate that Cas9 protein can efficiently cut DNA at the target site with direction of guide RNA and HDR can occur efficiently at the broken DNA site when a homologous DNA template is present within the cells (Fig. 3C).

To examine expression of PTEN‐long in the transfected and untransfected cells, Western blot analysis was employed to detect PTEN/PTEN‐long protein with a specific anti‐PTEN antibody as described above. PTEN‐long can only be detected in cells bearing the mutation CTG to ATG at the target site, and endogenous PTEN‐long was not detectable. The two guide RNAs had similar effects on directing HDR, regardless of whether they were transfected individually or jointly transfected into cells (Fig. 4A). As a positive control, cells transfected with the pcDNA PTEN‐long expression vector exhibited a band at ~75 kD protein that can be detected with the anti‐PTEN antibody (Fig. 4). Expression of PTEN‐long protein driven by CRISPR/Cas9 has the same molecular size as pcDNA PTEN‐long vector expressing PTEN‐long. It is noteworthy that homology DNA template is necessary for the correct repairing of the double‐stranded break of DNA in cells. In the absence of this DNA template, PTEN‐long was not detected in the cells transfected with either CRISPR/Cas9 vector bearing the guide RNA or only with single‐stranded DNA template (Fig. 4A). In contrast, the canonical PTEN is detected by Western blot analysis in these untransfected/control cells. PTEN‐long from PTEN‐long‐expressing cells is detectable in concentrated (×50) culture medium by Western blot analysis (Fig. 4B), indicating the PTEN‐long can be exported into culture medium.

**Figure 5:**
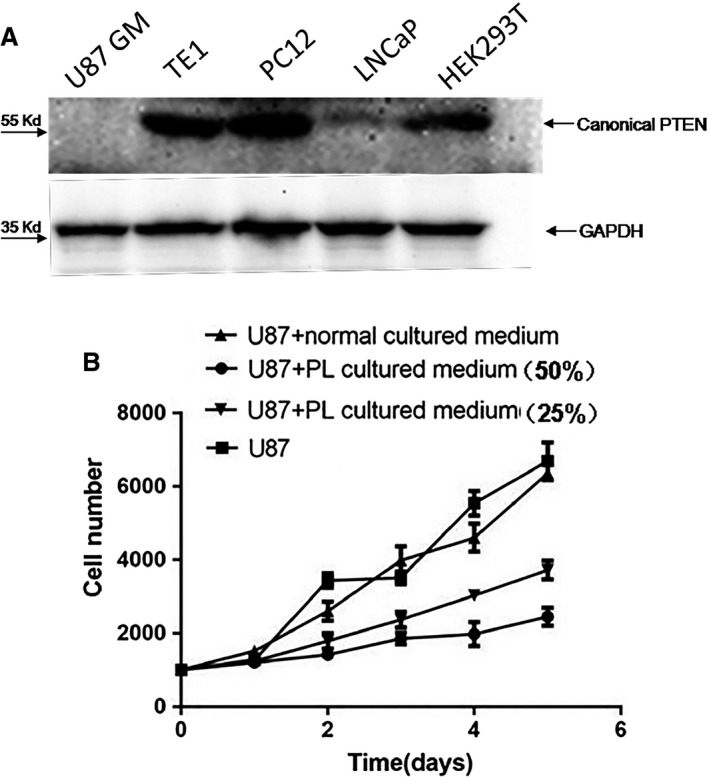
The PTEN‐long containing cultured medium suppresses PTEN‐null U87 cell proliferation. **A**. Five cell lines are identified for PTEN expression. U87 GM cells lack PTEN and LNCaP cells express PTEN protein weakly compared to TE1, PC12 and HEK293T cells. **B**. Growth curve of U87 cells in normal cultured medium or cultured medium from PTEN‐long‐expressing cells. Compared with normal U87 cells or cells growing in normal untransfected cultured medium, proliferation of U87 cells was down‐regulated by PL (PTEN‐long) cultured medium which account for 50% or 25% of total U87 growth medium. PTEN‐long expression is driven by CRISPR/Cas9, indicating that expressed PTEN‐long is secreted into the cultured medium and repress U87 cell proliferation.

Secreted PTEN‐long has previously been demonstrated to be biologically active, where it inhibited PI3K‐Akt signalling pathway and suppressed cancer cell proliferation and cell invasiveness in a similar manner to canonical PTEN 18, 28. In the present study, PTEN‐long expression in HEK293T cells was mediated by CRISPR/Cas9 combined with a homologous DNA template. To examine whether the secreted PTEN‐long had similar biological function as canonical PTEN, culture media from PTEN‐long‐expressing HEK293 cells were collected and concentrated (×50). The culture media were transferred to U87 cells, and then, the cell lysate was probed with anti‐PTEN antibody. The result shows the PTEN‐long can enter U87 cells (Fig. 4C) compared with control cell lysate. Subsequently, PTEN‐long activity was detected by determining Akt phosphorylation level and result indicated PTEN‐long reduced Akt phosphorylation compared with control medium or normal cells. In order to avoid interference of endogenous PTEN, we chose to use U87 cells, a PTEN‐null cell line (Fig. 5A) to examine effects of PTEN‐long on cell proliferation. As described above, 50% and 25% of PL cultured medium were added into U87 cell culture media. The cells in different groups were collected every 24 hrs over 5 days, and data were plotted using GraphPad Prism 5 GraphPad Software, Inc. (La Jolla, CA, USA). Results show that the cell culture medium from the cells highly expressing PTEN‐long can suppress proliferation of U87 cells in a dose‐dependent manner, compared with U87 cells cultured in normal media or with untransfected control culture media (Fig. 5B).

## Discussion

Development of tools for gene therapy for *in vivo* and *in vitro* use has been pursued for several decades and has primarily involved delivery of a therapeutic genetic material (DNA or RNA) to cells to augment a deficiency in the endogenous gene expression. However, for successful implementation of gene therapy, there are several hurdles to overcome. These include the nature of the micro‐environment *in vivo*, the difficulty in overcoming cell membrane barriers and the efficiency/specificity of delivering a therapeutic material to target cells (for review of current status of gene therapy see 29, 30, 31). In theory, a therapeutic protein can be directly delivered to target cells to augment loss of protein function either *via* the circulatory system or *via* injection at a local site. However, it is known that most proteins cannot freely enter cell membrane or tissues, unlike DNA or RNA which can be randomly delivered to cells with help of lipids and liposome. In addition, therapeutic proteins also have problems to overcome including instability, protein aggregation and immunogenicity, which has limited their utility 32, 33. Recently, nano materials have been developed as carrier systems to efficiently deliver therapeutic proteins to cells *in vivo* 34. However, more research is needed to determine their feasibility and specificity.

To overcome some of the above problems associated with gene therapy, CRISPR/Cas9 gene editing tool, was also carried out to edit a gene at genomic level for gene therapy 35, such that target genes could be repaired or deleted in genome. CRISPR/Cas9 has attracted increasing attention since the report of its successful use in mammalian cells 36, 37 or human cells 37, 38. This approach has made gene editing easier and more efficient than the previous gene editing tools, such as zinc finger nuclease (ZFN) and transcription activator‐like effector nucleases (TALEN) 39. In the present study, we designed two guide RNAs inserted into GeneArt CRISPR Nuclease Vector and both of which mediate cleavage sites followed by PAM close to the CTG on the *PTEN* loci (Fig. 1A). To confirm that a full‐length PTEN mRNA can be transcribed and appropriately spliced, and whether coding region of PTEN‐long protein is present within the mature mRNA, we performed a PCR amplification with primers covering the entire coding region of PTEN‐long, with the upstream primer carrying a mutation of CTU to ATG. Our results show a specific band generated in PCR from HEK293 cell genome with an expected correct size (Fig. 1B). Subsequently, the cDNA was cloned and verified by Sanger DNA sequencing. Data suggest that the full‐length PTEN mRNA contains the PTEN‐long coding region present in the HEK293 cells.

Following cotransfection of the recombinant vector and single‐stranded DNA, transfection efficiency was determined by observing the fluorescent cells under the microscope (Fig. 2). To easily track orange fluorescence protein and ensure the enzyme activity of Cas9 is not impacted, a self‐cleaving site 2A was inserted between Cas9 and orange fluorescence protein in a bicistronic manner. Genomic DNA from both untransfected and transfected cells were extracted and amplified by PCR as described in Materials and methods. Amongst the ten clones that were isolated, three carried the mutation of CTG to ATG, indicating the CRISPR/Cas9 plus single‐stranded DNA is able to induce a DNA break and subsequent DNA repair within the transfected cells. The target CTG codon remained unchanged in 70% cells as indicated from sequencing results, suggesting the DNA is either from untransfected cells or from the transfected cells in which CRISPR/Cas9 and the single‐stranded DNA were not efficiently cotransfected, or that either party did not work, as was previously reported 36.

Genomic editings mediated by the two guide RNAs in our experiments were similar to each other in fluorescence images and Western blot results (Figs 2B and D,  4A). The two guide RNAs also show similar efficiency in driving PTEN‐long expression either individually or when combined (Fig. 4A). It is evident that the enzyme Cas9 and the single‐stranded DNA template are required for PTEN‐long expression in the cells. In comparison with the PTEN‐long expression induced by transfection of pcDNA PTEN‐long, CRISPR/Cas9‐mediated expression of PTEN‐long is weaker. However, the advantage of CRISPR/Cas9‐mediated PTEN‐long expression is that it would be *in vivo* and the edited cells could permanently express PTEN‐long protein. To detect the capacity of PTEN‐long to be secreted, cell‐cultured media from edited cells were collected and concentrated by ultrafiltration centrifugation (×50). In Western blot analysis, we could observe PTEN‐long protein band from the concentrated cultured medium from the edited cell lysates, but not detectable from the original culture medium. This suggests that only a small quantity of endogenous PTEN‐long can be secreted into culture medium or that the secreted PTEN‐long protein is partially degraded in the culture medium, leading to little amount of PTEN‐long protein presence in the culture medium.

In the present study, we examined PTEN expression in a few cell lines and found that PTEN is completely deficient in U87 cells and only weakly expressed in LNCaP cells (Fig. 5A). The TE1, PC12 and HEK293T cells have normal PTEN protein expression bands in the Western blot results. To examine activity of the secreted PTEN‐long in the culture media, we collected edited cell culture media and transferred it to U87 cell culture, a PTEN‐null cell line. A previous report suggested that PTEN‐long has similar biological function in suppressing cancer cell proliferation *via* down‐regulating PI3K‐Akt signalling activity 18. We observed that PTEN‐long from the edited culture media can significantly suppress U87 cell proliferation compared to media from normal cultured cells or cells exposed to normal untransfected cell culture medium. We selected the U87 cells to examine cell proliferation curves, as these cells do not express PTEN and to avoid interference of endogenous PTEN in the cells. We observed that PTEN‐long can suppress U87 cell proliferation in a paracrine fashion in which PTEN‐long secreted in culture medium may enter neighbouring cells to suppress PI3K‐Akt signalling activity, leading to inhibition of cell proliferation. Hence, it is possible that for some cancer cells that do not express PTEN expression or harbour a gene mutation, PTEN‐long expression can be promoted from cells outside of the tumour location to induce tumour regression. The possibility of secreted PTEN‐long to permeate cancer cells and repress cancer proliferation has significant potential for therapy. Thus, the current investigation provides a novel approach to treat cancers in future.

## Conflict of interest

All authors declare that they have no conflict of interests with the contents of this article.
